# Combined massive allograft and intramedullary vascularized fibula transfer: the Capanna technique for treatment of congenital pseudarthrosis of the tibia

**DOI:** 10.1080/17453674.2020.1773670

**Published:** 2020-06-08

**Authors:** Stefanie C M Van Den Heuvel, Hay A H Winters, Klaas H Ultee, Nienke Zijlstra-Koenrades, Ralph J B Sakkers

**Affiliations:** a Amsterdam UMC, location VUmc;; b University Medical Center Utrecht, The Netherlands

## Abstract

Background and purpose — Congenital pseudarthrosis of the tibia (CPT) is caused by local periosteal disease that can lead to bowing, fracturing, and pseudarthrosis. Current most successful treatment methods are segmental bone transport and vascularized and non-vascularized bone grafting. These methods are commonly hampered by discomfort, reoperations, and long-term complications. We report a combination of a vascularized fibula graft and large bone segment allograft, to improve patient comfort with similar outcomes.

Patients and methods — 7 limbs that were operated on in 6 patients between November 2007 and July 2018 with resection of the CPT and reconstruction with a vascularized fibula graft in combination with a bone allograft were retrospectively studied. The mean follow-up time was 5.4 years (0.9–9.6). Postoperative endpoints: time to discharge, time to unrestricted weight bearing, complications within 30 days, consolidation, number of fractures, and secondary deformities.

Results — The average time to unrestricted weight bearing with removable orthosis was 3.5 months (1.2–7.8). All proximal anastomoses consolidated within 10 months (2–10). 4 of the 7 grafts fractured at the distal anastomosis between 6 and 14 months postoperatively. After reoperation, consolidation of the distal anastomosis was seen after 2.8 months (2–4). 1 patient required a below-knee amputation.

Interpretation — This case series showed favorable results of the treatment of CPT through a combination of a vascularized fibula graft and large bone segment allograft, avoiding the higher reintervention rate and discomfort with ring frame bone transport, and the prolonged non-weight bearing with vascularized fibula transfer without reinforcement with a massive large bone segment allograft.

Congenital pseudarthrosis of the tibia (CPT) is a rare disease affecting the development of the diaphysis of the tibia with a reported incidence ranging between 1 in 140,000 to 250,000 newborns (Ruggieri and Huson 2001, Horn et al. 2013). CPT is characterized by local periosteal disease, often leading to bowing and fracturing of the tibia and/or fibula, followed by the development of a pseudarthrosis (Stevenson et al. 1999). The etiology behind CPT remains largely unelucidated. Many theories regarding the influence of vascular, genetic, and mechanical factors have been proposed over the years, but none provides an entirely satisfactory explanation for the pathological features or its typical location (Hefti et al. 2000, Hermanns-Sachweh et al. 2005). However, there is a clear association with type 1 neurofibromatosis (NF1), as the prevalence of NF1 in CPT patients exceeds 50% (Van Royen et al. 2016).

The challenge in cases of pseudarthrosis in CPT is obtaining solid union of the tibia with minimal limb length discrepancy and angular deformity (Grill et al. 2000). The most used treatments today are resection of the CPT part of the bone and vertical bone transport or the use of a pedicled or free vascularized fibula graft (Kesireddy et al. 2018). A multinational study from Japan, which included both the Ilizarov technique combined with diaphyseal transfer and vascularized fibula grafting, found high rates of union among both treatment groups and concluded that both approaches should be considered (Ohnishi et al. 2005).

For the Ilizarov technique with diaphyseal transfer through proximal metaphyseal corticotomy and distraction at a distance from the dystrophic area, success rates between 50% and 90% have been reported (Paley et al. 1992, Ghanem et al. 1997, Romanus et al. 2000, Choi et al. 2011). Drawbacks of this technique are multiple interventions and prolonged discomfort of the patient due to many months of wearing a ring frame around the lower leg with pins and K-wires moving through muscle compartments. Also, pin tract infections and surgery to induce healing at the docking side of the bone transport lead to restrictions in social and psychological functioning (Ramaker et al. 2000, Patterson 2006).

Studies on vascularized fibula grafts report union rates up to over 90% (Weiland et al. 1990, Erni et al. 2010). Drawbacks of this method include the prolonged period of non-weight bearing due to the lack of primary mechanical strength, resulting in graft fractures requiring reoperations prior to consolidation and hypertrophy of the graft (Weiland et al. 1990, Bos et al. 1993, Romanus et al. 2000, Ohnishi et al. 2005).

In 1993, Capanna et al. reported on his “Capanna technique” in the resection of bone tumors in which the use of a vascularized autograft is combined with the use of a solid bone segment allograft in order to achieve instant stability with solid bone during consolidation and subsequent hypertrophy of the vascularized fibula during growth (Capanna et al. 1993).

In order to avoid the drawbacks and discomfort of bone transport or vascularized fibula transfer without adding initial additional stability, we introduced this technique for the treatment of pseudarthrosis in CPT. Paley (2019) recently published the outcomes of his cross-union concept, reporting union in all 17 treated patients without refracturing with his latest technique, with follow-up to 11 years. If these outcomes prove to be reproducible, this will probably make the cross-union technique the gold standard for treating CPT. We report a retrospective case series on the Capanna technique in patients with CPT as reference for future strategies in this disease.

## Patients and methods

All 6 patients operated on in the University Medical Center Utrecht between November 2007 and July 2018 with resection of a congenital pseudarthrosis of the tibia and immediate reconstruction with a vascularized fibula and a solid segment bone allograft were retrospectively reviewed. Baseline characteristics were obtained from hospital charts and included age, sex, Paley classification of CPT, data on patient history (prior interventions, type 1 neurofibromatosis), affected side, and donor side. The study was performed in accordance with the STROBE statement.

### Outcome measurements

Outcome measurements were: time to discharge, time to unrestricted weight bearing with a removable orthosis, complications within 30 days, radiological consolidation (consolidation of 3 cortices on the AP and lateral radiograph) of the proximal and distal anastomosis, number of fractures, time to fracture, final alignment, and limb-length discrepancy.

### Surgical technique

**Figure 1.**
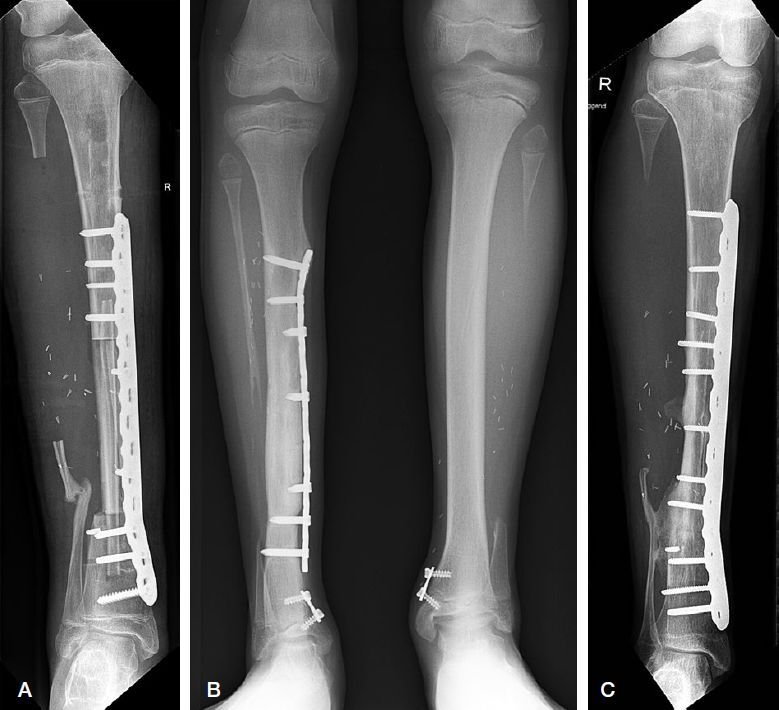
A. Postoperative image after Capanna procedure for CPT. B. 8-plates on both distal tibias to correct for ankle valgus at the donor and receptor sites. C. Cross-union between the distal anastomosis of graft and tibia and the remnant distal fibula.

The affected tibia was exposed through an anteromedial incision and wide resection of the affected segment of the tibia with periosteum was performed, including all hamartomatous tissue. The fibula was harvested in a standard fashion for free or pedicled transplantation through a lateral approach (At 1999, Stevanovic et al. 1999). The use of bone morphogenic protein (BMP) was not included in the present treatment protocol. The harvested vascularized fibula was placed intramedullary in the segmental tibial defect. If necessary, reaming of the intramedullary cavity was done. After secure intramedullary placement of the fibula graft, the microvascular anastomoses were performed when using a free contralateral fibula. The allograft (Bonebank ETB-BISLIFE, Leiden, the Netherlands) was cut to the appropriate length to bridge the gap between proximal and distal tibia. A vertical slot was cut in the allograft allowing it to be placed around the fibula, leaving the vascular pedicle of the fibula in the slot. Fixation of the allograft was done with a large LCP plate for instant stability (Figure 1A). After closure of the surgical incisions, the leg was placed in a lower-leg cast for 6 weeks. After 6 weeks a walking brace was applied at least until consolidation was observed on follow-up imaging. All patients received antibiotic prophylaxis and patients 3 and 6 additionally received thromboprophylaxis.

In the first 3 unilateral cases, we used the contralateral healthy fibula as a free vascularized graft. After having to use pedicled ipsilateral transplantation in a patient with bilateral CPT and encouraged by the results reported by Tan et al. (2011), ipsilateral transplantation became first choice in the last 2 patients of this case series with unilateral CPT.

### Ethics, funding, and potential conflicts of interest

The study received a “non-WMO” declaration from the Medical Research Ethics Committee of the University Medical Center Utrecht. No funding was obtained for this study and the authors have no conflicts of interest to declare.

## Results (Table 1)

### *Study population (**Table 2**)*

7 limbs were operated in 5 male patients (1 unilateral CPT left tibia, 4 unilateral CPT right tibia), and 1 female patient (bilateral CPT). The patient with bilateral CPT had Paley type 4A of the right lower leg and Paley type 4B of the left lower leg. The unilateral CPTs were one Paley type 1, one Paley type 3, and three Paley type 4A. Median age at the time of treatment was 5.5 years (3.4–16.5). Type 1 neurofibromatosis was present in 5 of the 6 patients.

3 patients (patients 1, 3, and 6) had been previously operated on unsuccessfully with cancellous bone grafting and placement of intramedullary rods.

The contralateral fibula was harvested and used as a vascularized free fibula in the reconstruction of 3 tibias. The ipsilateral proximal fibula was used as a pedicled fibula in the reconstruction of 4 tibias (3 patients). The dimensions of the allografts were length 10 cm (6.6–12) and width (medial–lateral) 1.6 cm (1–2.3) and (anterior–posterior) 2.4 (range 1.4–3.3).

### *Postoperative outcomes (**Table 3**)*

Follow-up was between 0.9 and 9.6 years. Average time to discharge following the combined vascularized fibula and bone allotransplantation was 5 days (3–6). None of the patients suffered a complication within the first 30 days following the procedure. Average time to unrestricted weight bearing with a removable orthosis was 3.5 months (1.2–7.8). Consolidation of the proximal anastomosis was seen after a mean period of 6 months (2–10). 4 of the 7 grafts fractured at the distal anastomosis between 6 and 14 months postoperatively. After reoperation, consolidation of the distal anastomosis was seen after 2.8 months (2–4). One tibia fractured distally to the anastomosis 6 years after reconstruction.

In all 3 patients who had the vascularized fibula from the healthy contralateral side, a valgus deformity of the distal tibia developed due to the hypotrophy of the lateral side of the distal epiphysis. 2 of these 3 patients also developed a valgus in the distal tibia of the acceptor limb due to hypotrophy of the distal epiphysis. A valgus deformity of the proximal tibia occurred in one patient. The limb-length discrepancy at the latest follow-up was 11 mm (0–44). 1 patient (procedures 3 and 4) was followed to skeletal maturity (mean 13.6 years, 8–19).

### Additional procedures

No additional operations were needed for the union of the proximal anastomosis and only an average of 0.71 (0–1) additional procedures were needed for union of the distal anastomosis. The majority of the other procedures were guided-growth procedures in day care.

Patient 1, who had a contralateral vascularized fibula transfer, developed a bilateral valgus deformity in the epiphysis of the distal tibia. Both sides were treated with an 8-plate over the medial side of the epiphyseal plate 4 years after the initial procedure (Figure 1B). 3 years later, the 8-plates were removed and a distal epiphysiodesis was performed on the donor site to minimize the discrepancy in leg length.

Patient 2, who had a contralateral vascularized fibula transfer, underwent a second procedure 3 years after the initial procedure during which the LCP plate was removed because the end of the curve of the plate deviated from the bone due to growth of the distal tibia. Additionally, an 8-plate was placed over the distal tibial epiphyseal plate to treat a valgus deformity of the distal epiphysis of the tibia. Progressive migration of the distal fibula with instability of the ankle joint was seen, which was treated by repositioning of the distal fibula with a Taylor spatial frame and subsequent creation of a synostosis of the distal tibia and distal fibula. In addition, the 8-plate on the distal medial side of the left tibia was replaced as the screws had developed maximum divergence and an 8-plate was placed on the distal medial side of the right tibia (donor site) to treat the valgus of the ankle on the donor side.

Patient 3 had bilateral ipsilateral vascularized fibula transfers. The distal anastomosis of the left tibia fractured after 15 months. Fusion by creating a synostosis between the distal tibia and distal remnant fibula segment was not successful at the first attempt and the patient chose to have an amputation and below-knee prosthesis. Her mobility with the below-knee prosthesis increased to such a level that she fractured the earlier operated right leg at the level of the distal screw of the LCP plate. The fracture healed after removing the LCP plate, drilling both distal tibia and fibula, and using a large spongiosa graft from a Reamer Irrigator Aspirator (RIA; DePuy Synthes, Warsaw, IN, USA) procedure from the ipsilateral femur. Latest follow-up showed a bony crossover between the distal tibia and the distal fibula.

In patient 4 (graft no. 5) a fracture of the distal anastomosis occurred 9 months after surgery. The fracture was treated with removal of the pseudarthrotic tissue, cancellous bone grafting from the iliac crest, and insertion of an intramedullary Rush nail resulting in successful consolidation. 3 years later, an 8-plate over the proximal tibial epiphyseal plate was placed to treat a valgus deformity.

Patient 5 had a fracture of the distal anastomosis of the graft and tibia 8 months after surgery. The fracture was treated with removal of the pseudarthrotic tissue and bone grafting from the iliac crest. Bone healing occurred after 8 weeks with a synostosis between the distal anastomosis of the tibia and the remnant distal fibula (Figure 1C).

Patient 6 (graft no. 7) had a fracture at the distal anastomosis between graft and tibia 6 months after surgery. The fracture was treated with replacement of the LCP plate and an RIA procedure of the femur to harvest spongiosa bone for cross-union between the distal anastomosis of the tibia and the remnant fibula.

## Discussion

This case series reports on the Capanna technique using a large segment of bone allograft in combination with a vascularized fibula graft after resecting part of the tibia with CPT. The technique was used in order to improve on the current techniques by avoiding the discomfort of external frames for bone transport, or prolonged non-weight bearing when using vascularized fibular grafts without additional bony stabilization.

It has been well documented that external fixation in children and adolescents has a significant physical and physiological impact, with studies reporting pain and consequent sleeping problems in approximately half of the patients (Ramaker et al. 2000). The complications related primarily to the use of an external device and include pin-track infection, loosening, breakage, refracture at the pin insertion site, neurovascular injury, axial deviation, osteopenia, and joint stiffness. Residual limb-length discrepancy and valgus deformity are commonly reported with an overall complication rate of 30–100% (Choi et al. 2011). In addition, a comprehensive review on the physiological effects of external fixation demonstrated depression to be universally evident to varying degrees (Patterson 2006). Standardized and validated quality of life measurements were not obtained in this retrospective study. However, we can reasonably assume that the minor discomfort of a lower leg cast does not have the reported outcomes associated with external fixation.

As compared with the technique of vascularized fibula grafts without the support of a massive allograft, the time to full-weight bearing has been reported to be as long as 18–24 months (Kalra and Agarwal 2012). Gilbert (1983) extended this period to up to 3 years, and Weiland et al. (1990) required their patients to wait until skeletal maturity was reached. In this study combining vascularized fibula grafts with a massive allograft, full weight bearing with a removable orthosis was already possible after 1.2 to 7.8 months. The significantly shortened time to weight bearing associated with the present technique underlines the benefits of combining the VFG with the massive allograft, particularly in this young cohort of patients.

One of the undesired side effects that was seen in 3 patients who had the vascularized fibular graft taken from the healthy contralateral side was the development of hypotrophy of the lateral side of the distal epiphysis of the tibia and subsequent valgus of the ankle joint. Valgus deformities at the donor site were also reported in previous studies (Weiland et al. 1990, Ohnishi et al. 2005). The compensatory asymmetric growth on the metaphyseal side of the growth plate, induced by the 8-plate, compensated for the valgus deformity at the epiphyseal side of the growth plate.

The undesirable side effect of hypotrophy of the distal epiphysis with subsequent valgus of the ankle joint in the healthy leg and the publication by Tan et al. (2011) made us switch from the contralateral to the ipsilateral donor leg, and thereby from free vascularized to a pedicled segment of the fibula. Obviously, the amount of available healthy fibula is likely to be smaller in the ipsilateral leg, as the fibula is also affected by the disease in most cases. This must be assessed, and if there is not enough healthy bone in the ipsilateral fibula a free vascularized graft from the contralateral leg is still indicated. In case of a pedicled fibula, the pedicle can be distally or proximally based depending on the position of the defect, the quality of the vascular pedicle, and the position of the healthy segment of the fibula.

The most important complication was the fracturing of the site of the distal anastomosis in 4 out of 7 grafts. Even a vascularized fibula bridging the distal anastomosis and stabilization with massive allograft and large fragment LCP plate does not always create adequate circumstances for primary solid bony fusion at this location. Therefore, we changed the protocol and added the creation of a synostosis between the distal anastomosis and the remnant of the distal fibula with a spongiosa graft from the ipsilateral femur to the initial procedure. Although slightly different from the crossover technique by Paley (2019), we hope this will primarily strengthen the distal anastomosis and prevent fractures.

The fracture that appeared 6 years after the initial surgery was just distal of the most distal screw of the LCP plate. The question therefore arises as to whether removal of the LCP plate after successful consolidation would have prevented this fracture and should become standard protocol.

The use of bone morphogenic protein (BMP) was not included in the present treatment protocol, because the use of BMP in children is off-label in Europe.

All patients in our cohort underwent 1 or more additional procedure(s) during follow-up. No additional operations were needed for the union of the proximal anastomosis and only an average of 0.71 (0–1) additional procedures were needed for union of the distal anastomosis. Most other procedures were guided-growth procedures in day care to correct for a valgus deformity in the upper ankle joint. This is in line with previous studies on the use of vascularized fibula grafts in CPT patients showing residual valgus deformities in up to 80% of patients in the upper ankle joint (Weiland et al. 1990, Ohnishi et al. 2005). Previous studies involving free vascularized fibula grafts reported reintervention rates between 37% and 60% (Bos et al. 1993, Romanus et al. 2000).

The results of the European Paediatric Orthopaedic Society multicenter study by Grill et al. (2000) reported 340 patients who underwent 1,287 procedures (meaning 3.79 interventions per patient) and a fusion rate of 76%.

In summary, the Capanna technique for ipsilateral vascularized fibular transplantation needed only 1.2 interventions to achieve union in 6 out of 7 tibias with a relative short time to full weight bearing and seems therefore to be a more patient-friendly treatment than the conventional methods of bone transport with ring frames or vascularized fibula transfers that do not use the addition of a massive allograft in the reconstruction. We hope that the outcomes will be further improved by adding the creation of a synostosis between the distal anastomosis and the remnant of the distal fibula with a spongiosa graft from the ipsilateral femur to the initial procedure.

**Table 2.** Baseline characteristics

Procedure Patient		Age (years)	NF	Side	Paley classification	Previous procedures	Donor site
1	1	5.5	Yes	Right	4A	4	Contralateral
2	2	3.4	Yes	Left	1	0	Contralateral
3	3 ^a^	12.7	Yes	Right	4A	1	Ipsilateral
4	3 ^a^	16.5	Yes	Left	4B	0	Ipsilateral
5	4	3.0	Yes	Right	4A	0	Contralateral
6	5	5.1	Yes	Right	3	0	Ipsilateral
7	6	14.0	No	Right	4A	1	Ipsilateral

**^a^**Bilateral

**Table 1.** Results

Procedures	Patients	Primary proximal union	Refracture proximal	Primary distal union	Refracture distal
7	6	7	0	3	4

**Table 3.** Postoperative outcomes

Procedure Patient		Follow-up (years)	LOS ^a^ (days)	Consolidation proximal part tibia	Months to consolidation proximal	Months to consolidation distal	Compli- cation < 30 days	Complication 1 > 30 days	Complication 2 > 30 days
1	1	9.6	5	Yes	8	13	–	Bilateral valgus deformity ankle	Leg length discrepancy
2	2	9.4	6	Yes	5	13	–	Bilateral valgus deformity ankle	Leg length discrepancy
3	3	7	6	Yes	5	4 **^b^**	–	Fracture distal tibia	
4	3	3	3	Yes	10	– **^c^**	–	Fracture distal tibia and fracture plate	Pseudarthrosis requiring amputation
5	4	4.1	6	Yes	2	2 **^b^**	–	Persisting distal Pseudarthrosis	Valgus deformity proximal tibia
6	5	3.7	5	Yes	7	2 **^b^**	–	Fracture distal tibia	
7	6	0.9	4	Yes	5	3 **^b^**	–	Fracture distal tibia	

**^a^** Length of stay in hospital. **^b^** Consolidation after additional procedure. **^c^** Below-knee amputation after refracture.
